# Article-Level Metrics and the Evolution of Scientific Impact

**DOI:** 10.1371/journal.pbio.1000242

**Published:** 2009-11-17

**Authors:** Cameron Neylon, Shirley Wu

**Affiliations:** 1 Science and Technology Facilities Council, Rutherford Appleton Laboratory, Didcot, United Kingdom; 2 23andMe, Mountain View, California, United States of America

## Abstract

The authors discuss the value of article-level metrics in determining an article's scientific impact.

Formally published papers that have been through a traditional prepublication peer review process remain the most important means of communicating science today. Researchers depend on them to learn about the latest advances in their fields and to report their own findings. The intentions of traditional peer review are certainly noble: to ensure methodological integrity and to comment on potential significance of experimental studies through examination by a panel of objective, expert colleagues. In principle, this system enables science to move forward on the collective confidence of previously published work. Unfortunately, the traditional system has inspired methods of measuring impact that are suboptimal for their intended uses.

## Measuring Impact

Peer-reviewed journals have served an important purpose in evaluating submitted papers and readying them for publication. In theory, one could browse the pages of the most relevant journals to stay current with research on a particular topic. But as the scientific community has grown, so has the number of journals—to the point where over 800,000 new articles appeared in PubMed in 2008 (http://www.ncbi.nlm.nih.gov/sites/entrez?Db=pubmed&term=2008:2008[dp], archived at http://www.webcitation.org/5k1cbn1WX on 24 September 2009) and the total is now over 19 million (http://www.ncbi.nlm.nih.gov/sites/entrez?Db=pubmed&term=1800:2009[dp], archived at http://www.webcitation.org/5k1crb7Pi on 24 September 2009). The sheer number makes it impossible for any scientist to read every paper relevant to their research, and a difficult choice has to be made about which papers to read. Journals help by categorizing papers by subject, but there remain in most fields far too many journals and papers to follow.

As a result, we need good filters for quality, importance, and relevance to apply to scientific literature. There are many we could use but the majority of scientists filter by preferentially reading articles from specific journals—those they view as the highest quality and the most important. These selections are highly subjective but the authors' personal experience is that most scientists, when pressed, will point to the Thomson ISI Journal Impact Factor [1] as an external and “objective” measure for ranking the impact of specific journals and the individual articles within them.

Yet the impact factor, which averages the number of citations per eligible article in each journal, is deeply flawed both in principle and in practice as a tool for filtering the literature. It is mathematically problematic [2]–[4], with around 80% of a journal impact factor attributable to around 20% of the papers, even for journals like *Nature* [5]. It is very sensitive to the categorisation of papers as “citeable” (e.g., research-based) or “front-matter” (e.g., editorials and commentary) [6], and it is controlled by a private company that does not have any obligation to make the underlying data or processes of analysis available. Attempts to replicate or to predict the reported values have generally failed [5]–[8].

Though the impact factor is flawed, it may be useful for evaluating journals in some contexts, and other more sophisticated metrics for journals are emerging [3],[4],[9],[10]. But for the job of assessing the importance of specific papers, the impact factor—or any other journal-based metric for that matter—cannot escape an even more fundamental problem: it is simply not designed to capture qualities of individual papers.

## Article-Level Metrics

If choosing which articles to read on the basis of journal-level metrics is not effective, then we need a measure of importance that tells us about the article. It makes sense that when choosing which of a set of articles to read, we should turn to “article-level metrics,” yet in practice data on individual articles are rarely considered, let alone seriously measured.

Perhaps the main reason for this absence is a practical one. Accurately determining the importance of an article takes years and is very difficult to do objectively. The “gold standard” of article impact is formal citations in the scholarly literature, but citation metrics have their own challenges. One is that citation metrics do not take the “sentiment” of the citation into account, so while an article that is heavily cited for being wrong is perhaps important in its own way [11], using citation counts without any context can be misleading. The biggest problem, though, is the time-delay inherent in citations. The first citations to a paper will appear—at the earliest—months after it is first available; far too late to be useful in the days and weeks after it is published. If we are looking for a way to evaluate recently published papers of potential interest to us, citation-based metrics are not the answer.

## The Trouble with Comments

A common solution proposed for getting rapid feedback on scientific publications is inspired by the success of many Web-based commenting forums. Sites like Stack Overflow, Wikipedia, and Hacker News each have an expert community that contributes new information and debates its value and accuracy. It is not difficult to imagine translating this dynamic into a scholarly research setting where scientists discuss interesting papers. A spirited, intelligent comment thread can also help raise the profile of an article and engage the broader community in a conversation about the science.

**Figure 1:**
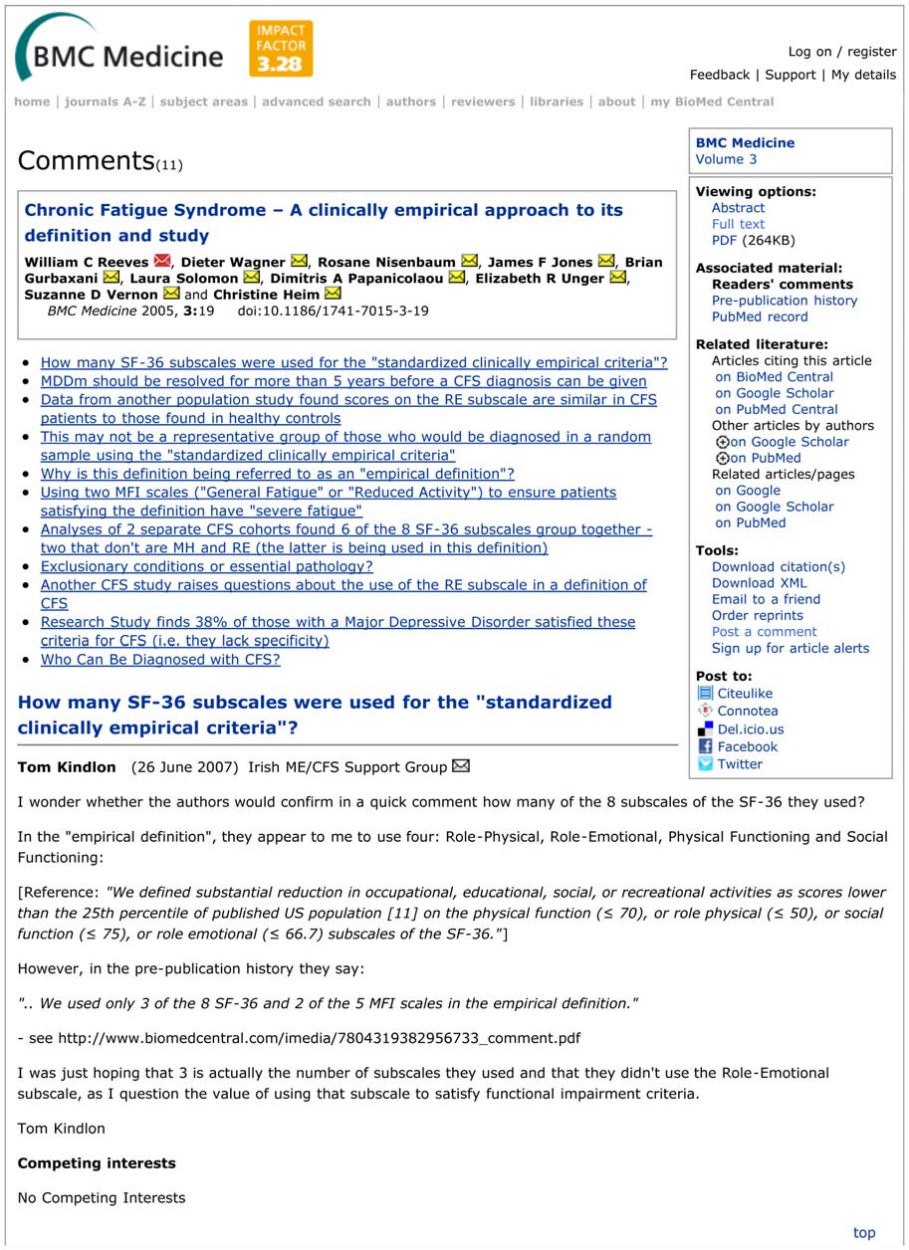
Examples of article comments on BioMed Central. The comments shown are for an article published in BMC Medicine [14]. Full names and affiliations are typically given and commenters also indicate conflicts of interest. Readers must also be logged in to comment. Note that conversations are not threaded—meaning that replies cannot be formally directed towards specific comments—though commenters sometimes indicate whether their comment pertains to a specific comment via the “re:” designation in the comment title.

**Figure 2:**
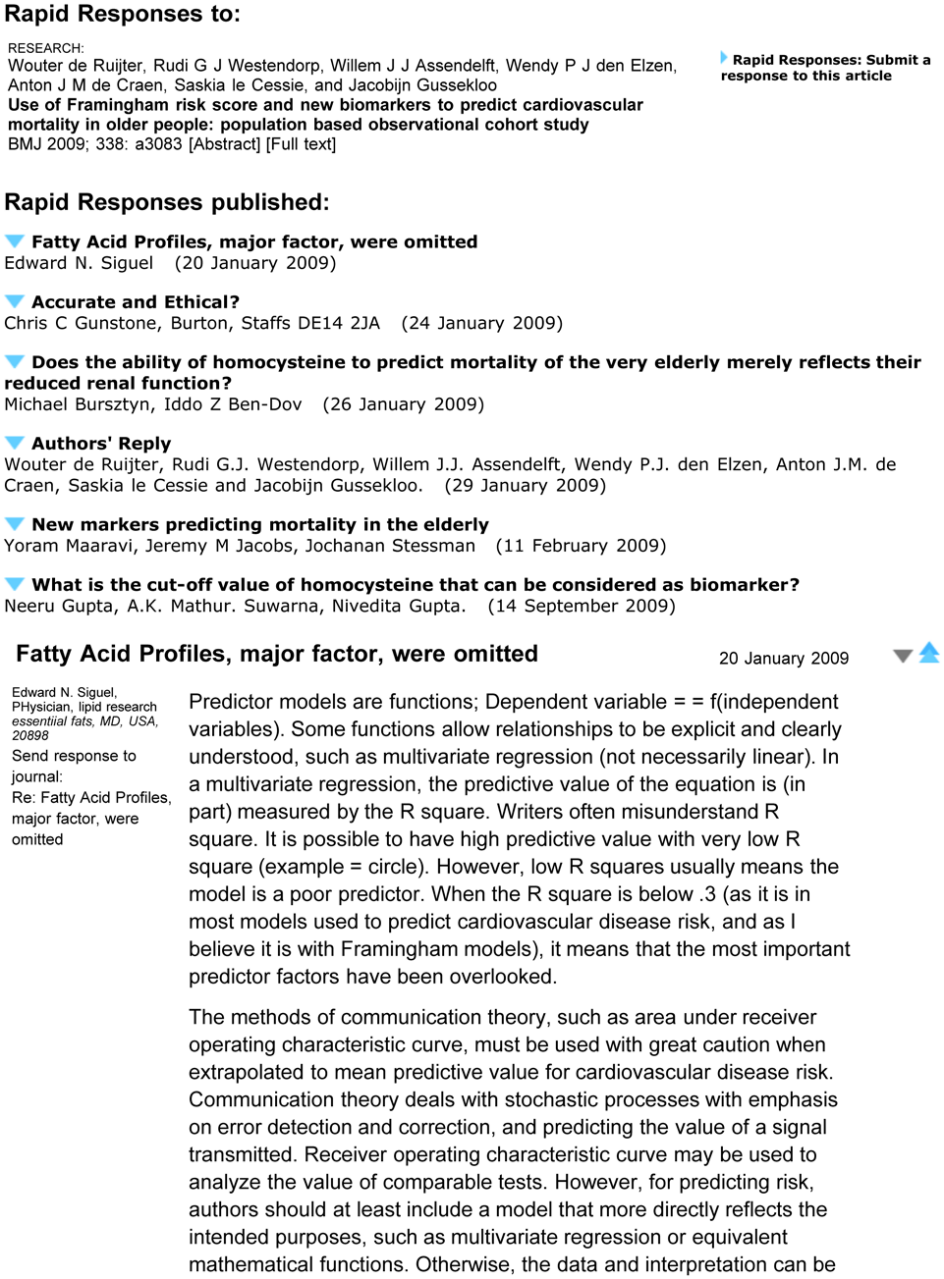
Examples of article comments on *BMJ*. The comments shown are for a research article published in early 2009 [17]. Full names and affiliations are typically given, and commenters indicate conflicts of interest but no login is needed to comment. As with BMC articles, threading is not formally implemented.

Unfortunately, commenting in the scientific community simply hasn't worked, at least not generally. BioMedCentral, PLoS, and BMJ have all had commenting platforms for several years (see Figures 1 and 2 for examples), and while certain papers have extensive discussions [12]–[15], these are the exception rather than the rule. Moreover, highly commented papers tend to fall under the category of “front matter” rather than primary research (e.g., a recent Perspective article in *PLoS Biology* had 17 comments and over 20,000 page views on 4 October 2009 [16]). Attempts to apply a “Digg-like” mechanism for voting up or down on the basis of perceived value—on the ArXiv preprints service, for instance—have failed to gain traction [17]. At the same time new sites based on similar systems have flourished for communities as diverse as programmers and amateur knitters. Why is community participation apparently easy for some but not for researchers—whose work, after all, depends on constructive feedback from their peers?

Part of this resistance to commenting may relate to technical issues, but the main reason is likely social. For one thing, researchers are unsure how to behave in this new space. We are used to criticizing articles in the privacy of offices and local journal clubs, not in a public, archived forum. Junior researchers may be concerned about the potential repercussions on their own careers. Anonymity can support more direct and honest discussion but as anyone who has visited popular video or news sites knows, anonymity often degrades discussions to the point where they detract from the original piece.

Another issue is that the majority of people making hiring and granting decisions do not consider commenting a valuable contribution. Stack Overflow, which caters to programmers, works in part because its contributors build up “karma” through a points-based system that translates into greater influence within the community. More importantly, high Stack Overflow karma can be taken beyond the site to add credibility to your resume and directly enhance career opportunities. There is currently no analogous credit system for post-publication commenting in the scientific community. And if there is no reward for quality contribution then people will struggle to justify the time involved in generating high quality comments. It is interesting in this sense that the one commenting system that does appear to obtain reasonable amounts of researcher input is the Faculty of 1000, where senior researchers are selected to become “Faculty” and contribute their opinions on papers they believe are important. Being able to place “Member: Faculty of 1000” on your CV is incentive enough to encourage contributions of sufficient quantity and quality.

Then there is simply the size of the community. There are many more people broadly involved in programming (or knitting) than there are scientists. For community-based social media efforts, there is a well-known 90-9-1 rule: 90% of people merely observe, 9% make minor contributions, and 1% are responsible for the vast majority of original content (http://www.90-9-1.com/). This breakdown need not be a bad thing—on any given article you want the people who care and who have the expertise to be providing critical commentary. But it also means that if only 100 people read a paper, it will be lucky if even one of them leaves a comment.

## Technical Solutions to Social Problems

Given the lack of incentive, are there ways of capturing article-level metrics from what researchers do anyway? A simple way of measuring interest in a specific paper might be via usage and download statistics; for example, how many times a paper has been viewed or downloaded, how many unique users have shown an interest, or how long they lingered. This method can certainly provide a rapid means of assessing interest in a paper by comparing the trend in downloads and page views against the average. A common objection is that these statistics can be artificially inflated by faking or automating downloads, but this problem has been largely solved by the online advertising industry, which relies on trusted page view and download metrics provided by third parties. These statistics may not be completely accurate but they are consistent, comparable, and considered sufficiently immune to cheating to be the basis for a billion dollar Web advertising industry.

A more important criticism of download statistics is that it is a crude measure of actual use. How many of the downloaded papers are even read, let alone digested in detail and acted upon? What we actually want to measure is how much *influence* an article has, not how many people clicked on the download button thinking they “might read it later.” A more valuable metric might be the number of people who have actively chosen to include the paper in their own personal library. Endnote, Refworks, and libraries in BibTex format have been the traditional tools for managing personal reference libraries, but there is a growing set of tools with some significant advantages: the tools are free, easy to use, and can help to provide high value article-level metrics without requiring any additional effort on the part of the researchers.

Examples of such tools are Zotero, Citeulike, Connotea, and Mendeley, which all allow the researcher to collect papers into their library while they are browsing on the Web, often in a single click using convenient “bookmarklets.” The user usually has the option of adding tags, comments, or ratings as part of the bookmarking process. From this point on the tools differ in a variety of ways: Zotero and Mendeley allow formatting citations within manuscripts, whereas Citeulike and Connotea are more focused on using tags and feeds to share information. Importantly, however, they all provide information on *how many people have bookmarked a specific paper*. Citeulike, Connotea, and Zotero currently go further by providing information on precisely *who* has bookmarked a specific paper, a piece of information that may be very valuable, although people can choose not to make that information public. Some of these tools may also eventually be able to track the amount of time users spend viewing papers within their interface [18].

Metrics collected by reference management software are especially intriguing because they offer a measure of active interest without requiring researchers to do anything more than what they are already doing. Scientists collect the papers they find interesting, take notes on them, and store the information in a place that is accessible and useful to them. A significant question is why would they share that valuable information with the rest of the world? The data would still be useful even without identities attached, but researchers are more likely to share openly if appropriate incentive structures exist, as in the example of Faculty of 1000.

Part of the solution to encouraging valuable contributions, then, may simply be that the default settings involve sharing and that people rarely change them. A potentially game-changing incentive, however, may be the power to influence peers. By broadcasting what papers they think are important, researchers are directly influencing the research community's choice of reading and discussion material. This type of influence can be both a good thing, in providing the type of recognition that drives career prospects and will enhance the quality of contributions, but also potentially bad in as much as it concentrates power in the hands of the few. In a sense it is a shift in power from one set of editors, those currently in charge of journals, to a new set of “editors” who curate published papers in a different, but possibly just as useful way.

It is too early to tell whether any specific tools will last, but they already demonstrate an important principle: a tool that works within the workflow that researchers are *already using* can more easily capture and aggregate useful information. With researchers constantly pressured for time and attention, approaches that gather information from processes that are already part of the typical research workflow are also much more likely to succeed.

## The Great Thing about Metrics…Is That There Are So Many to Choose From

**Figure 3:**
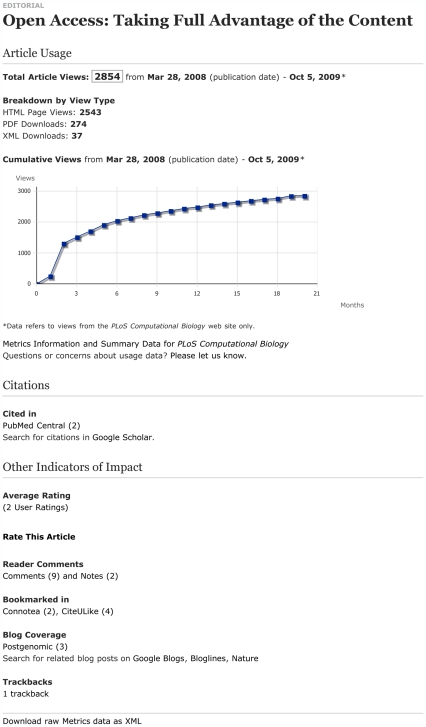
Article-level metrics provided by PLoS. Shown here are article-level metrics for a recent article in *PLoS Comput Biol* [15], accessible by clicking the “Metrics” tab on the article Web page. At the top, page view and download counts provide an immediate measure of the number of times people accessed the article via the journal Web site. The plot showing cumulative views gives a sense of the trend in views over time. Citations are shown next and refer to the number of other articles that have referenced this one as indexed in citation databases; PubMed Central and Google Scholar in this case. Citations typically take months or years to appear, but are better indicators of how many other articles have built upon the work. “Other Indicators of Impact” include ratings and comments, which, like page views, are immediate but may offer more insight because users are more likely to have read the article and found it compelling enough to respond. Additional other indicators are bookmarks, used by some people to keep track of articles of interest to them, and blog posts and trackbacks, which indicate where else on the Web the article has been mentioned and can be useful for linking to a broader discussion. It is clear that all of the types of data provide different dimensions, which together can give a clearer picture of an article's impact.

There are numerous article-level metrics (see Figure 3) and each has its own advantages and problems. Citation counts are an excellent measure of influence and impact but are very slow to collect. Download statistics are rapid to collect but may be misleading. Comments can provide valuable and immediate feedback, but are currently sparse and require a change in the research reward culture to become more widespread and to improve quality. Bookmarking statistics can be both rapid to collect and contain high quality information but are largely untested and require the widespread adoption of unfamiliar tools. Alongside these we have “expert ratings” by services such as Faculty of 1000 and simple rating schemes.

The metrics are also useful in different contexts. Indeed, the fundamental problem of which paper to read can also have different contexts. Which new papers are relevant to you? Which papers should you read if you are going to pursue research question X? Which papers do you need to read before submitting your paper? Are you a funder interested in media coverage of work you have paid for or a textbook writer aiming to assess the most important contributions in a field? All of these questions require different information at different times and may be better determined using different measures of article impact. As recently shown [10], scientific impact is not a simple concept that can be described by a single number. The key point is that *journal* impact factor is a very poor measure of *article* impact. And, obviously, the fact that an article is highly influential by any measure does not necessarily mean it should be.

Many researchers will continue to rely on journals as filters, but the more you can incorporate effective filtering tools into your research process, the more you will stay up-to-date with advancing knowledge. The question is not whether you should take article-level metrics seriously but how you can use them most effectively to assist your own research endeavours. We need sophisticated metrics to ask sophisticated questions about different aspects of scientific impact and we need further research into both the most effective measurement techniques and the most effective uses of these in policy and decision making. For this reason we strongly support efforts to collect and present diverse types of article-level metrics without any initial presumptions as to which metric is most valuable. Different users will need different information and it will take time before any metric proves useful.

As Clay Shirky famously said [19], you can complain about information overload but the only way to deal with it is to build and use better filters. It is no longer sufficient to depend on journals as your only filter; instead, it is time to start evaluating papers on their own merits. Our only options are to publish less or to filter more effectively, and any response that favours publishing less doesn't make sense, either logistically, financially, or ethically. The issue is not how to stop people from publishing, it is how to build better filters, both systematically and individually. At the same time, we can use available tools, networks, and tools built on networks to help with this task.

So in the spirit of science, let's keep learning and experimenting, and keep the practice and dissemination of science evolving for the times.
